# A novel *LRP6* variant in a Japanese family with oligodontia

**DOI:** 10.1038/s41439-021-00162-w

**Published:** 2021-07-20

**Authors:** Hiroki Goto, Masashi Kimura, Junichiro Machida, Akiko Ota, Mitsuko Nakashima, Naomi Tsuchida, Junya Adachi, Yoshihiko Aoki, Tadashi Tatematsu, Katsu Takahashi, Masatoshi Sana, Atsuo Nakayama, Shintaro Suzuki, Toru Nagao, Naomichi Matsumoto, Yoshihito Tokita

**Affiliations:** 1 Department of Maxillofacial Surgery, School of Dentistry, Aichi-Gakuin University, Nagoya, Japan; 2 Department of Oral and Maxillofacial Surgery, Toyokawa City Hospital, Toyokawa, Japan; 3 Department of Disease Model, Institute for Developmental Research, Aichi Developmental Disability Center, Kasugai, Japan; 4 Department of Oral and Maxillofacial Surgery, Ogaki Municipal Hospital, Ogaki, Japan; 5 Department of Human Genetics, Yokohama City University Graduate School of Medicine, Yokohama, Japan; 6 Department of Oral and Maxillofacial Surgery, Toyota Memorial Hospital, Toyota, Japan; 7 Department of Oncology, Toyota Memorial Hospital, Toyota, Japan; 8 Department of Stem Cell and Immune Regulation, Yokohama City University Graduate School of Medicine, Yokohama, Japan; 9 Dentistry and Oral surgery Tazuke Kofukai, Medical Research Institute, Kitano Hospital, Osaka, Japan; 10 Nagoya Orthodontic Clinic, Nagoya, Japan; 11 Department of Cellular Pathology, Institute for Developmental Research, Aichi Developmental Disability Center, Kasugai, Japan

**Keywords:** Disease genetics, Development

## Abstract

Congenital tooth agenesis is a common anomaly in human development. We performed exome sequence analysis of genomic DNA collected from Japanese patients with tooth agenesis and their relatives. We found a novel single-nucleotide insertion in the *LRP6* gene, the product of which is involved in Wnt/β-catenin signaling as a coreceptor for Wnt ligands. The single-nucleotide insertion results in a premature stop codon in the extracellular region of the encoded protein.

Tooth agenesis is one of the most common developmental anomalies in humans; however, its underlying cause is complex and remains largely unknown. We have previously reported that the prevalence rates of hypodontia and oligodontia, which are subtypes of the condition, in the Japanese population are 6.8% and 0.1%, respectively; we also found that sibling recurrence risks corresponded to 25.0% and 43.8%, respectively. These observations suggest that oligodontia, the more severe tooth agenesis phenotype of the two subtypes, is mostly inherited in a dominant manner^1^. Multiple congenitally missing teeth have been associated with abnormalities in several genes encoding Msh homeobox 1^2–5^, ectodysplasin A^6,7^, axin inhibition protein 2^8^, paired box gene 9^9^, Wnt family member 10A (*WNT10A*)^10,11^, and low-density lipoprotein receptor-related protein 6 (*LRP6*).

To date, 12 functionally null variants of the *LRP6* gene have been identified in families with congenital tooth agenesis^12–15^. In this study, we performed whole-exome sequencing (WES) as previously described^16^ using genomic DNA from a Japanese family with congenital tooth agenesis and identified a novel frameshift mutation in *LRP6*. This study was approved by the Aichi-Gakuin University Committee on the Ethics of Human Experimentation (Nagoya, Japan; approval no. 58), the Institute for Developmental Research (Kasugai, Japan; approval no. 13-07) and IRB of Yokohama City University Graduate School of Medicine (Yokohama, Japan, approval no. A201200014).

A 48-year-old Japanese woman diagnosed with familial oligodontia was referred to our hospital (II-2, Fig. 1A). Clinical examination, including radiographic analysis, confirmed the diagnosis of oligodontia in the proband with 23 missing permanent teeth (Fig. 1B). Subsequently, three other members of her family (I-1, II-1, and III-3) were analyzed, and one of her children (III-3) was diagnosed with oligodontia. However, we could not definitively diagnose her father with congenital tooth agenesis because of severe tooth loss. All of the family members had normal primary dentition, nails, skin, and hair.

**Fig. 1:**
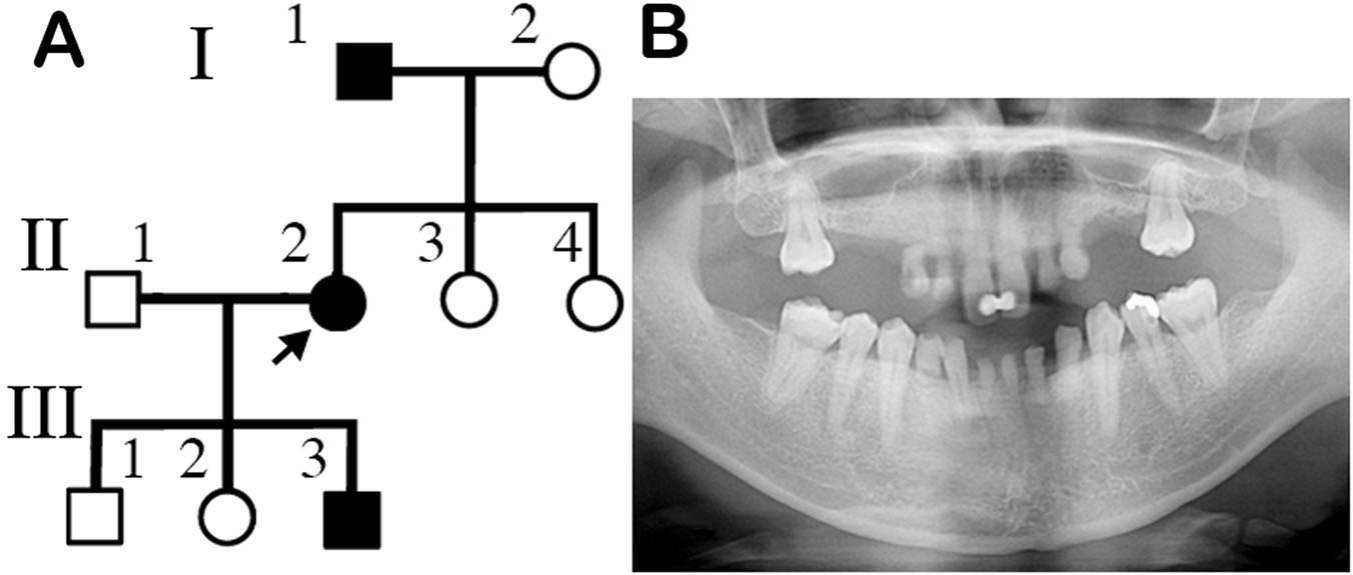
Pedigree of the case patients and permanent tooth number anomalies in the proband. **A** Family pedigree. The arrow indicates the proband (II-2). Open square, nonaffected male; solid square, affected male; open circle, nonaffected female; solid circle, affected female. **B** Panoramic radiograph of the proband (II-2) showing the absence of permanent teeth.

WES detected a heterozygous single-nucleotide insertion in exon 9 of *LRP6* (NM_002336.2(LRP6_v001): c.1924dup) (Fig. 2). The variant is not present in Exome　Aggregation Consortium or the 1000 Genome database. Polymerase chain reaction and Sanger sequencing confirmed that our index case patient’s child, III-3, and father, I-1, also carried this insertion. The mutation is predicted to generate a protein with a truncated extracellular domain (NM_002336.2(LRP6_i001): p.(p.Ile642Asnfs11*)).

**Fig. 2:**
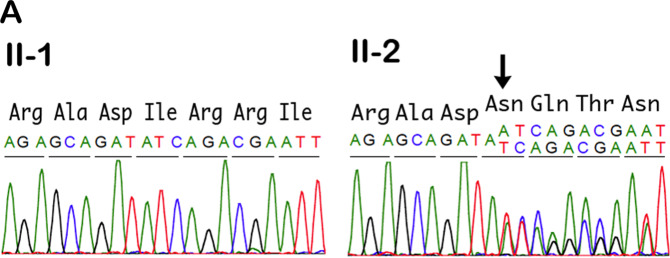
*LRP6* (low-density lipoprotein receptor-related protein 6 gene) mutation cosegregates with tooth agenesis. **A** Partial DNA sequence of exon 9 (nucleotide position, 1915–1936) of the *LRP6* gene (NM_002336.2) in a nonaffected family member (II-1; Left panel). Heterozygous mutation in the proband (II-2; Right panel). The single base insertion (arrow) leads to an amino acid substitution of Ile to Asn at 642 and introduces a premature stop codon after an unrelated 11-amino acid sequence.

The Wnt family consists of 19 secreted glycoproteins that regulate homeostasis in various adult tissues and are related to various diseases via the actions of several signaling molecules, such as frizzled receptors and LRP coreceptors^17^. *WNT10A* is the causative gene for rare autosomal recessive ectodermal dysplasia presenting with syndromic tooth agenesis^18,19^. Disfunction of WNT10A is associated not only with recessive syndromic congenital anomalies but also with a dominantly inherited form of selective tooth agenesis^20^. The *LRP5* and *LRP6* genes are crucial for various Wnt/β-catenin signaling pathways. Several studies have revealed that loss-of-function mutations in *LRP6* cause the autosomal dominant form of selective tooth agenesis (STHAG7: MIM 616724)^12–15^. Therefore, the Wnt/β-catenin signaling pathway is considered to be associated with tooth development.

In conclusion, we identified a novel *LRP6* variant, c.1924dup, in a Japanese family with tooth agenesis. The gene product of the p.(p.Ile642Asnfs11*) variant is a truncated form of the protein lacking transmembrane and cytoplasmic domains. Moreover, we attempted to screen shared gene variations among case patients using WES data, but promising candidate genes other than *LRP6* variant were not identified. Therefore, c.1924dup appears to be a novel causative variant for tooth agenesis in the family investigated. Further studies are needed to determine the precise molecular pathology of Wnt-signaling-related diseases.

## HGV database

The relevant data from this Data Report are hosted at the Human Genome Variation Database at 10.6084/m9.figshare.hgv.3078.

## References

[1] Machida J (2014). Genetic epidemiology of tooth agenesis in Japan: a population- and family-based study. Clin. Genet..

[2] Vastardis H (1996). A human MSX1 homeodomain missense mutation causes selective tooth agenesis. Nat. Genet..

[3] Kamamoto M (2011). Clinical and functional data implicate the Arg(151)Ser variant of MSX1 in familial hypodontia. ur. J. Hum. Genet..

[4] Yamaguchi S (2014). Characterization of novel MSX1 mutations identified in Japanese patients with nonsyndromic tooth agenesis. PLoS ONE.

[5] Tatematsu T (2015). An aberrant splice acceptor site due to a novel intronic nucleotide substitution in MSX1 gene is the cause of congenital tooth agenesis in a Japanese family. PLoS ONE.

[6] Shufeng L (2008). Non-syndromic tooth agenesis in two Chinese families associated with novel missense mutations in the TNF Domain of EDA (Ectodysplasin A). PLoS ONE.

[7] Han D (2008). Novel EDA mutation resulting in X-linked non-syndromic hypodontia and the pattern of EDA-associated isolated tooth agenesis. Eur. J. Med. Genet..

[8] Lammi L (2004). Mutations in AXIN2 cause familial tooth agenesis and predispose to colorectal cancer. Am. J. Hum. Genet..

[9] Jumlongras D (2004). A novel missense mutation in the paired domain of PAX9 causes non-syndromic oligodontia. Hum. Genet..

[10] Bohring A (2009). WNT10A mutations are a frequent cause of a broad spectrum of ectodermal dysplasias with sex-biased manifestation pattern in heterozygotes. Am. J. Hum. Genet..

[11] Machida J (2017). WNT10A variants isolated from Japanese patients with congenital tooth agenesis. Hum. Genome Var..

[12] Massink MP (2015). Loss-of-function mutations in the WNT co-receptor LRP6 cause autosomal-dominant oligodontia. Am. J. Hum. Genet..

[13] Ockeloen CW (2016). Novel mutations in LRP6 highlight the role of WNT signaling in tooth agenesis. Genet. Med..

[14] Yu M (2021). Lrp6 dynamic expression in tooth development and mutations in oligodontia. J. Dent. Res..

[15] Brance ML (2020). High bone mass from mutation of low-density lipoprotein receptor-related protein 6 (LRP6). Bone.

[16] Sakamoto M (2021). Novel EXOSC9 variants cause pontocerebellar hypoplasia type 1D with spinal motor neuronopathy and cerebellar atrophy. J. Hum. Genet..

[17] Clevers H (2006). Wnt/beta-catenin signaling in development and disease. Cell.

[18] Adaimy L (2007). Mutation in WNT10A is associated with an autosomal recessive ectodermal dysplasia: the odonto-onycho-dermal dysplasia. Am. J. Hum. Genet..

[19] Cluzeau C (2011). Only four genes (EDA1, EDAR, EDARADD, and WNT10A) account for 90% of hypohidrotic/anhidrotic ectodermal dysplasia cases. Hum. Mutat..

[20] van den Boogaard MJ (2012). Mutations in WNT10A are present in more than half of isolated hypodontia cases. J. Med. Genet..

